# Polymorphism of Methylenetetrahydrofolate Reductase (MTHFR) gene and risk of Head and Neck Squamous Cell Carcinoma

**DOI:** 10.1590/S1808-86942010000600017

**Published:** 2015-10-19

**Authors:** Juliana Olsen Rodrigues, Ana Lívia Silva Galbiatti, Mariangela Torreglosa Ruiz, Luiz Sergio Raposo, José Victor Maniglia, Érika Cristina Pavarino-Bertelli, Eny Maria Goloni-Bertollo

**Affiliations:** 1 Medical student; 2 Biologist, master's degree student in Health Sciences; 3 Doctoral degree in Health Sciences; 4 Physician, master's degree student in Health Sciences; 5 Adjunct professor; 6 Associate professor in Human Genetics, adjunct professor; 7 Associate professor in Human Genetics, adjunct professor. FAMERP - Faculdade de Medicina (medical school) de São José do Rio Preto

**Keywords:** genes, head and neck neoplasms, polymorphism

## Abstract

**Abstract:**

Methylenetetrahydrofolate reductase gene (MTHFR) C677T polymorphism may be a risk factor for head and neck squamous cell carcinoma due to changes in folate levels that can induce disorders in the methylation pathway, which results in carcinogenesis.

**Aim:**

To evaluate MTHFR C677T polymorphism in patients with head and neck squamous cell carcinoma and in individuals with no history of cancer, and to assess the association of this disease with clinical histopathological parameters.

**Series and Methods:**

A retrospective study that assessed gender, age, tobacco, alcohol consumption and clinical histopathological parameters in 200 patients (100 with disease and 100 with no history of cancer). PCR-RFLP molecular analysis was carried out and the chi-square test and multiple logistic regression were applied for the statistical analysis.

**Results:**

There was no association between MTHFR C677T polymorphism and head and neck cancer (p = 0.50). Significant differences between the study and control groups were observed at age over 50 years, tobacco use, and male gender (p <0.001). There was no association of disease with clinical-histopathological parameters.

**Conclusion:**

No association between the MTHFR C677T polymorphism and head and neck squamous cell carcinoma was possible in this study.

## INTRODUCTION

Head and neck squamous cell carcinoma is one of the five most common types of cancer worldwide. It is sixth in cancer mortality and comprises cancers of the mouth (40%), pharynx (15%), and larynx (25%).^1^

Head and neck squamous cell carcinoma patients are generally diagnosed at a late stage of the disease; about 75% of cases are diagnoses in stages III or IV. The situation is worse in developing countries, such as India, Thailand and Brazil,^3^ where this cancer is more common.^4^ The mean survival rate in these patients is 5 years.^5^ The 5-year life expectancy is about 50% when there are lymph node metastases.^6^, ^7^ No significant increase in survival rates had occurred in the last few decades in spite of new surgical techniques, radiotherapy and concomitant chemotherapy.^4^, ^5^, ^6^, ^8^

Epidemiologic studies have suggested a multifactor etiology for this cancer; these predisposing factors include smoking, alcohol use, human papilloma virus (HPV) infection, and genetic factors.^1^, ^2^, ^3^, ^4^, ^7^, ^9^, ^10^

Genetic polymorphisms leading to folate deficiency appear to facilitate the onset and growth of head and neck squamous cell carcinoma and other types of cancer. Folate is part of DNA methylation, in which methyl groups (CH3) are transferred to the 5’ position of cytosine residues on cytosine-guanine (CpG) dinucleotides in reactions catalyzed by proteins (DNA methyltransferases).^11^ This epigenetic modification of DNA has several functions, such as controlling gene expression, stabilizing the chromatin structure, and maintaining genomic stability.^11^, ^12^, ^13^, ^14^, ^15^, ^16^, ^17^, ^18^, ^19^, ^20^, ^21^, ^22^

C677T polymorphism of the methyenetetrahydrofolate reductase (MTHFR) gene appears to be related with cancer susceptibility because the activity of the MTHFR enzyme (which is part of folate metabolism) is reduced; this may cause uncontrolled gene expression, genomic instability, and induce carcinogenesis.^23^, ^24^

Because of the significant ethnic and geographic variation in the frequency of the C677T variant of the MTHFR gene, researchers have evaluated the Hardy-Weinberg equilibrium of this gene in different populations to study whether allele frequencies remain unaltered across generations. Jim et al.'s meta-analysis (2009)^25^ investigated five Asian and five European or American ethnic populations and confirmed that the genotype distribution in the control group in two studies did not abide by the Hardy-Weinberg principle. Studies by Suzuki et al. (2007),^15^ Solomon et al. (2008),^26^ and Kruszyna et al. (2009),^27^ respectively in Asian, Polish and Indian populations, found that genotype frequencies were in Hardy-Weinberg equilibrium.

Based on current evidence, the purpose of this study was to identify MTHFR C677T polymorphism in patients with head and neck squamous cell carcinoma and in subjects with no history of cancer, and to investigate associations between this polymorphism, clinical findings, demographic data (age and gender) and risk factors (smoking habits and alcohol consumption) in this disease.

## SERIES AND METHODS

The institutional review board of the institution approved this study (number 5566/2005).

There were 200 subjects in this study; 100 (84 male and 16 female; mean age - 59.05 ± 10.4 years) were patients with a histopathologic diagnosis of head and neck squamous cell carcinoma, seen at a university hospital in the Northeastern part of São Paulo state, and 100 other subjects (76 male and 24 female; mean age - 43.61 ± 2.2 years) that had no medical history of cancer. Subjects were included in the study after signing a free informed consent form.

The control group included individuals from the blood bank of São José de Rio Preto, with no diagnosis of cancer according to the blood donor protocol that assesses 20 diseases (http://www.hemonline.com.br/portarias/rdc153/indexframe.htm). Another inclusion criterion was age over 40 years; exclusion criteria were a family history of cancer and the presence of any disease in the blood donor protocol. The study group consisted of patients with a diagnosis of head and neck carcinoma based on pathology specimens from biopsies or total excision. The inclusion criterion was patients with the squamous cell histological type; previously treated patients were excluded.

The study variables were age, gender, exposure to risk factors (alcohol consumption and cigarette smoking), and tumor-related clinical parameters. Smokers were defined here as subjects that had smoked about 100 cigarettes in their lives, and alcohol consumers were subjects that drank more than four drinks a week.^28^, ^29^ The clinical parameters included the primary sites of tumors (mouth, pharynx and larynx); tumors were classified according to the parameters issued by the Union International Control Cancer (IUCC), 2002, and the American Joint Committee for Cancer (AJCC), 2002, as follows: tumor size (T), and presence of involved regional lymph nodes (N). Clinical and pathology data were gathered from medical registries.

Extracted genomic DNA, according to Miller et al. (1998), modified,^30^ was PCR-amplified, according to Yi et al., 2002.^31^ The amplification product was digested with the Hinf I restriction enzyme according to the manufacturer's instructions, to detect the polymorphic site (MTHFR 677T).

The multiple logistic regression model was applied to evaluate the effect of variables and the genotype distribution among groups. The model included the following variables: age (reference: age below the median in both groups), gender (reference: female sex), smoking habits (reference: non-smokers); alcohol consumption (reference: non-drinkers).

Multiple logistic regression was also applied to analyze the clinical features and pathology. The T classification was the same in small (T1, T2) and large tumors (T3, T4). The N classification was separated into negative (N0) and positive lymph node involvement (N1, N2, N3).

Results are presented as odds ratio (OR) and 95% confidence intervals (CI - 95%). The significance level was 5% (p=0.05). The Bioestat and Instat software packages were used in the statistical analysis.

## RESULTS

Table 1 presents the social and demographic data of 100 patients with head and neck squamous cell head and neck squamous cell carcinoma and of 100 subjects with no history of cancer. There were significant differences between the study and control groups at ages over 50 years, smoking habits, and male gender (p < 0.001). There were 160 male subjects (80%; 84 patients and 76 controls) and 40 female subjects (20%; 16 patients and 24 controls). Smokers comprised 88% of the study group, compared to 43% in the control group. Alcohol consumers were 77% of the study group and 49% of controls. There was no significant difference between these groups (p=0.06).

**Table 1:** Distribution of demographic data, risk factors, MTHFR C677T genotype and the odds ratio (OR) patients with head and neck squamous cell carcinoma and subjects with no history of cancer

Variables	Patients (n = 100) %	Controls (n = 100) %	OR (IC 95%)	p
Age				
mean (years)	59,05	43,61		
< 50 years	19	79	Reference	< 0.001
> 50 years	81	21	20,81 (9.01-48.09)	
Gender				
Female	16	24	Reference	0,02
Male	84	76	0,27 (0.09-0.82)	
Smoking				
No	88	43	Reference	0,001
Yes	12	57	5,03 (1.93-13.15)	
Alcohol consumption				
No	23	51	Reference	0,06
Yes	77	49	2,38 (0.94-6.05)	
C677T polymorphism				
CC	44	46	Reference	0,50
CT/TT	43/13	40/14	1,31 (0,59-2,93)	

* Adjusted for age, gender, tobacco and alcohol consumption (multiple logistic regression). There were statistically significant differences among groups for age over 50 years, male gender, and tobacco and alcohol consumption.

Smokers were subjects that consumed up to 5 cigarettes a day. Alcohol consumers were subjects that consumed on average up to 4 drinks a week (multiple logistic regression).

The Hardy-Weinberg equilibrium showed that genotype distributions were similar to those expected among patients (X^2^ = 1.18; p = 0.23) and in controls (X^2^ = 0.23; p = 0.63). The genotype CC, CT and CC frequencies of the C677T polymorphism were respectively 44%, 43% and 13% for patients, and 46%, 40% and 14% in controls. There were no significant differences in genotype distribution between groups (p= 0.92).

The allele frequencies for head and neck cancer patients were respectively, C=0.65 and T=0.35, and for controls C=0.66 and T=0.34. There were no statistically significant differences between groups (p=1.00).

The multiple logistic regression test was applied to evaluate the effect of variables on disease progression (genotypes, age, gender, smoking and alcohol consumption) (see Table 2). Age over 50 years (OR = 20.81; CI= 9.01 to 48,09; p < 0.0001) and smoking (OR= 5.03; CI =1.93 to 13.15; p < 0.001) were predictors of disease.

**Table 2:** Distribution of clinical and pathology parameters and MTHFR C677T polymorphism

Clinical findings*/ Genotypes	Frequency	OR (IC 95%)	p
Mouth			
MTHFR CC	14	Reference	0,51
MTHFR CT/TT	22	1,46 (0,47-4,55)	
Pharynx			
MTHFR CC	11	Reference	0,52
MTHFR CC/TT	11	0,722 (0,27-1,96)	
Larynx			
MTHFR CC	14	Reference	0,96
MTHFR CT/TT	17	1,02 (0,43-2,44)	
T1 e T2			
MTHFR CC	17	Reference	
MTHFR CT/TT	32		
T3 e T4			0,08
MTHFR CC	19	0,45 (0,19-1,10)	
MTHFR CT/TT	15		
N0			
MTHFR CC	29	Reference	
MTHFR CT/TT	37		0,62
N+			
MTHFR CC	9	1,36 (0,39-4,78)	
MTHFR CT/TT	15		

* Only tumors of the mouth, pharynx, and larynx were considered. The analysis was made only of patients with complete registries. MTHFR CT or TT genotypes compared with clinical parameters. Reference: MTHFR CC (multiple logistic regression)

Polymorphism was not associated with primary tumor sites (mouth, pharynx and larynx), tumor extension, and lymph node involvement. Primary sites were the mouth (34% of patients), the larynx (31%), and the larynx (22%). The remaining sites were unknown. The TNM classification was stages T1 or T2 in 59% of patients - of which 34,6% had the CC wild genotype, and 65.4% had at least one mutated allele (CT or TT genotypes). The remaining patients (41%) were stages T3 or T4, of which 55% had the CC wild genotype, and 45% had at least one mutated allele (CT or TT genotypes). Lymph nodes were involved (N+) in 26.6% of patients (37.5% with the CC genotype, and 62.5% with at least one mutated allele - CT or TT genotypes).

## DISCUSSION

This study showed that head and neck squamous cell carcinoma is more frequent in smokers aged over 50 years. Published results have also shown that this cancer is more frequent after the fifth decade of life and in smokers.^32^, ^33^, ^34^

No relationship between alcohol consumption and head and neck squamous cell carcinoma was found, such as in the study by Boccia et al. (2009).^35^ Animal studies have suggested that alcohol is not genotoxic, but that it may act as a solvent for the entry of carcinogens. Acetaldehyde, the primary metabolite of alcohol, is highly reactive and binds to proteins, cells substrates, and DNA, forming DNA adducts.^36^, ^37^, ^38^ Excessive alcohol consumption may also lead to nutritional deficiencies because of impaired intestinal absorption and altered metabolic pathways.^39^ The results of a multicenter study have suggested that in the absence of smoking, the association between alcohol consumption and head and neck carcinoma is insignificant; alcohol appears to be carcinogenic only at high doses.^40^

We found no association between gender and this disease in our series; other studies, however, have found this association and have suggested that the incidence of this disease in females has increased in the past few decades. Males are still more affected by this type of tumor.^41^, ^42^

MTHFR C677T polymorphism has been studied as a risk factor for cancer susceptibility because of its effect on cell methylation reactions and DNA synthesis. Global genome hypomethylation occurs with greater frequency in tumors compared to normal cells; it is thought that increased oncogene expression by result from hypomethylation. On the other hand, hypermethylation of CpG islands located in gene promoting regions has been associated with altered gene expression, resulting in tumor suppressing gene silencing.^43^

The genotype frequencies in our study were in Hardy-Weinberg equilibrium. The polymorphic genotype (MTHFR 677TT) was found in 14% of controls and 13% of patients with head and neck squamous cell carcinoma. There is considerable ethnic and geographic variation in the frequency of the C677T variant of the MTHFR gene. The prevalence of the MTHFR 677TT genotype ranges from 1% in blacks (US, Africa and South America) to over 20% in Europeans, Colombians, and American Indians; 12% of Japanese were TT homozygotes.^44^ Because of the ample mixture of races in the Brazilian population, no analysis of ethnic prevalence were made in our series.

We found no association between MTHFR C677T polymorphism and head and neck carcinoma in our series. Other studies have shown an association between this polymorphism and several cancers, such as those of the lung^45^, ^35^ liver (hepatocellular carcinoma),^46^ a few leukemias,^47^ prostate cancer,^48^ breast,^49^ colon,^49^ endometrium, esophagus, stomach, pancreas, and bladder.^46^ A meta-analysis of nine head and neck carcinoma studies found no association between this polymorphism and this disease, which concurs with our results.^32^ However, Reljic et al. (2007)^50^ and Solomon et al. (2008)^26^ found that head and neck carcinoma and the MTHFR C677T polymorphism were associated in Croat and Indian populations.

Reljic et al. (2007)^50^ studied 81 patients with head and neck carcinoma and 102 controls, and found a statistically significant difference in the genotype distribution of both groups, which provides evidence of a possible association between MTHFR C677T polymorphism and head and neck carcinoma. This analysis revealed that the 677CT genotype reduces the risk of this disease by 2.8 times. Solomon et al. (2008)^26^ studied 126 Indian subjects with mouth carcinoma (33 were alcohol consumers, 56 were moderate alcohol consumers, and 37 did not consume alcohol) and showed that alcohol consumers with the MTHFR 677TT genotype were at a higher risk for developing mouth carcinoma (OR - 3.0; 95% CI = 2.02-4.0) and that the TT genotype protected only non-alcohol and moderate alcohol consumers.

There are no published studies describing abnormalities of the gene above and head and neck squamous cell carcinoma in Brazil; although our results were not statistically significant, it is interesting to study these abnormalities because of contradictory results and the paucity of studies.

The most frequent tumor site was the mouth - 40% of head and neck cancers.^51^, ^52^ In the TNM classification, 19% of cases were stage T1, 30% were stage T2, 17% were stage T3, and 17% were stage T4. Lymph nodes were not involved in 73.4% of patients, and there were no metastases in 99% of cases. A retrospective Brazilian study revealed a high frequency of this disease at advanced stages.^53^ This discrepancy with our findings appears to reflect the type of study population; in our case, monitored patients.

MTHFR C677T polymorphism was not associated with the clinical parameters of this study (primary tumor sites, tumor extension, and lymph node involvement). Capaccio et al.^54^ (20050 studied the presence of this polymorphism in several oropharyngeal anatomical sites and found no association among the abovementioned variables. There was no association between this polymorphism and disease extension (T stage) and tumor staging in the three regions of the head and neck (mouth, pharynx, and larynx) when analyzing tumor aggressiveness, which concurs with Vairaktaris et al.'s (2006) study.^55^

## CONCLUSION

To conclude, no associations were found between MTHFR C677T polymorphism and head and neck squamous cell carcinoma. Our data showed that the frequency of this disease is higher after the fifth decade of life and in smokers. Clarifying any association between this polymorphism and head and neck carcinoma - and the association of this cancer type with other risk factors - will help understand the neoplastic mechanisms underlying this disease, as well as prevention and control measures.
